# Graphene Field Effect Transistors for Biomedical Applications: Current Status and Future Prospects

**DOI:** 10.3390/diagnostics7030045

**Published:** 2017-07-26

**Authors:** Rhiannan Forsyth, Anitha Devadoss, Owen J. Guy

**Affiliations:** Centre for Nanohealth, College of Engineering, Swansea University, Swansea SA2 8PP, UK; anitha.devadoss@swansea.ac.uk

**Keywords:** G-FET (graphene-based field effect transistors), DNA, aptamer, Debye length, antigen binding fragment, Dirac voltage, point-of-care

## Abstract

Since the discovery of the two-dimensional (2D) carbon material, graphene, just over a decade ago, the development of graphene-based field effect transistors (G-FETs) has become a widely researched area, particularly for use in point-of-care biomedical applications. G-FETs are particularly attractive as next generation bioelectronics due to their mass-scalability and low cost of the technology’s manufacture. Furthermore, G-FETs offer the potential to complete label-free, rapid, and highly sensitive analysis coupled with a high sample throughput. These properties, coupled with the potential for integration into portable instrumentation, contribute to G-FETs’ suitability for point-of-care diagnostics. This review focuses on elucidating the recent developments in the field of G-FET sensors that act on a bioaffinity basis, whereby a binding event between a bioreceptor and the target analyte is transduced into an electrical signal at the G-FET surface. Recognizing and quantifying these target analytes accurately and reliably is essential in diagnosing many diseases, therefore it is vital to design the G-FET with care. Taking into account some limitations of the sensor platform, such as Debye–Hükel screening and device surface area, is fundamental in developing improved bioelectronics for applications in the clinical setting. This review highlights some efforts undertaken in facing these limitations in order to bring G-FET development for biomedical applications forward.

## 1. Introduction

The discovery of Graphene in 2004 by Novoselov and Geim [1] brought with it many advances in scientific research. Graphene is a single-atom-thick carbon sheet with *sp^2^* bonded carbon arranged in a honeycomb structure. The unique properties of graphene, including excellent conductivity, rapid electron transport, large surface area, and biocompatibility [1,2], make it an attractive candidate for energy, environmental, and healthcare applications [3]. The development of the first enzyme-based biosensor by Clark and Lyons in 1962 [4] has resulted in vital biomedical devices, such as glucose biosensors [5]. Biosensors are essentially comprised of two main components; a biorecognition molecule (or capture molecule), and a signal transducer that determines the performance of the sensor. In the last several years, numerous studies have developed a wide range of biosensor systems and transduction techniques for the highly sensitivite detection of disease biomarkers. In particular, graphene biosensors represent a rapidly expanding multi-disciplinary field due to their higher sensitivity, wide linear detection ranges, and rapid detection, as the majority of disease biomarkers are typically present at ultra-low concentrations at the onset of the disease or illness [6]. For example, graphene-based biocatalytic sensors, such as enzyme biosensors, exhibit higher sensitivities owing to graphene’s excellent electronic conductivity. On the other hand, an affinity-based sensor, such as an immunosensor, utilizes a surface-immobilized recognition probe to selectively interact with the biological analyte in solution, and yields an electrical signal directly proportional to the analyte concentration.

Recent advances in the microfabrication techniques have led to the development of next-generation bioelectronic devices, including silicon nanowires [7,8,9,10], carbon nanotubes [11,12,13], and graphene-based field effect transistor (G-FET) devices for biosensor applications. This review particularly focuses on graphene-based field effect transistor devices because of their functionalizable surface and highly sensitive electronic properties. A G-FET is made up of a conducting graphene channel across two metal contacts, the source and drain electrodes, through which the current is conveyed. Here, the graphene is chemically functionalized with biomolecule receptors, such as antibodies or single-strand DNA probes, which can selectively bind to the target biomolecules in solution. The binding of target biomolecules to the graphene channel leads to a change in charge or electric potential at the G-FET surface, resulting in a charge carrier density and mobility variation within the G-FET, which leads to an electrical conductivity change associated with biomolecular binding events. Thus, the chemically modified G-FET device transduces the biological signal into an electrical signal at the bioelectronics interface upon each binding event [14]. Due to their ultrahigh mobility [15], G-FETs respond rapidly to variations of gate-source voltage [16], enabling a unique and powerful platform for detecting binding events.

G-FET biosensors are particularly attractive in point-of-care diagnosis due to their miniaturization, potential for large-scale manufacture at low-cost, rapid and inexpensive assays, and reduced need for skilled personnel. Moreover, G-FET biosensors offer the benefits of high sensitivity, lower detection limits, low cost, and high throughput detection compared to the existing enzyme-linked immunosorbent assay (ELISA), Polymerase Chain Reaction (PCR), and fluorescence methods, which are time consuming and require expensive and complex optical imaging devices and sophisticated image recognition software [16]. It is for these reasons that many G-FET biosensors have already been developed and reported in the literature. In fact, conducting a search on the NCBI Pubmed Central database using the words “graphene field effect transistors” flagged up 1501 entries. When widening this search to “graphene biosensors”, over 2400 entries appeared. Many of these G-FETs include pH sensors, enzyme-modified sensors, DNA-based sensors, and immunosensors [17].

This review is organized to emphasize the recent developments in affinity-based G-FET biosensors. We will briefly discuss the properties of graphene functionalization techniques in the context of bioelectronics in Section 2. Section 3 discusses affinity-based G-FET biosensors for the highly sensitive detection of biomolecules.

## 2. Graphene Platform

### 2.1. Graphene Properties

Graphene, or single atomic thick carbon, is the first purely two-dimensional (2D) material to be obtained [18]. Graphene is made up of carbon atoms which are bound to three others with a 120° bond angle, resulting in a hexagonal lattice arrangement of *sp^2^*-hybrised carbon [19]. The 2D nature and hexagonal carbon arrangement is the basis of graphene’s high specific surface area (2630 m^2^/g), a trait which is particularly advantageous in biosensing applications [20]. Graphene is considered attractive for electronic applications due to its intrinsically exceptional ballistic charge transport [18]. Experimentally, carrier mobilities have been reported to be about 2 orders of magnitude larger than the “gold-standard” semiconductor, silicon. Carrier mobilities have been known to exceed 10^7^ cm^2^·V^−1^·s^−1^ in graphene that has been decoupled from bulk graphite, to be as high as 10^5^ cm^2^·V^−1^·s^−1^ in suspended graphene devices [21], and about 4 × 10^3^ cm^2^·V^−1^·s^−1^ for CVD graphene on a SiO_2_ substrate. Moreover, graphene material can be manufactured in large quantities and relatively cheaply, therefore making it a suitable substrate for large-scale electronic device manufacturing [22].

**Figure 1:**
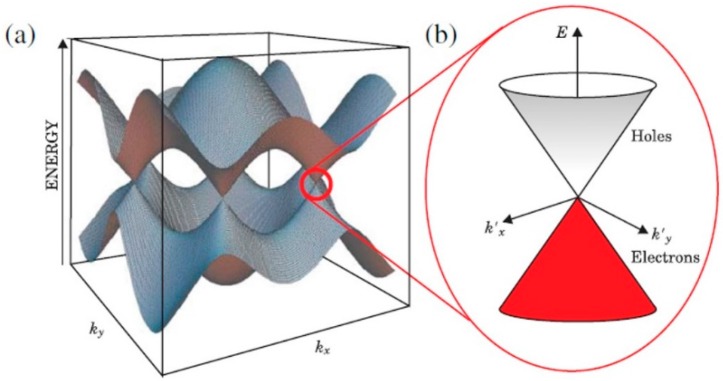
(**a**) Schematic representation of the conductance band (CB) and valence band (VB) meeting at the *K* and *K’* points of the Brillouin zone at the Fermi level. (**b**) A schematic representation of a Dirac cone showing in more detail the intersection of the VB and CB at the Fermi level. Adapted from [23]. Copyright 2011 by the American Physical Society.

Graphene consists of two energy bands, the valence band (VB) and the conductance band (CB), which hold holes and electrons, respectively [23]. The arrangement of the carbon atoms of graphene in a honeycomb lattice creates a completely full VB and an empty CB, as depicted in Figure 1 [19]. The two bands intersect at a point called a Dirac point, or the *K* and *K’* points in the Brillouin zone. At the point where they meet, depicted by the Dirac voltage (V_D_) in V_g_–I_DS_ measurements, the Fermi level passes across. This Fermi level can be tuned and adapted because of doping by external influences, such as electron deficient (p-doping) or electron rich (n-doping) molecules [18], therefore essentially causing a shift in the V_D_ to a more positive voltage (p-doping) or to a more negative voltage (n-doping). The V_D_ can therefore be monitored and utilized as a means of sensing biological molecules. The electronic properties, such as the V_D_, carrier mobility, and resistance, can be influenced by many external sources, these include: applying an electrical field, charged moieties near the graphene’s surface, or by chemically modifying the surface, such as chemical binding to the graphene both covalently and non-covalently [18].

### 2.2. G-FET Development

Graphene FETs are generally fabricated using micro fabrication techniques, such as photolithography coupled with metal evaporation or physical vapor deposition (PVD), to pattern and develop the device contacts. The graphene is either then transferred from a copper substrate used for its growth (CVD graphene) or from exfoliated graphene on to a patterned device [24]. Alternatively, a bulk graphene layer (CVD graphene on SiO_2_/Si or epitaxial graphene) is plasma etched away to form a channel [25]. Many G-FETs produced in this manner are highlighted in Table 1.

The channel is then modified to detect target biomarkers by immobilizing bioreceptors onto the graphene channel. This can be done directly (adsorption) or through a linker molecule. The immobilization of a highly specific bioreceptor (a process termed biofunctionalization) to the graphene surface induces chemical specificity towards the target biomarker. Such receptors may include amino acids, enzymes, antibodies, aptamers, or indeed any selective and specific molecule [26]. However, if a linker molecule is required, the graphene channel must first be chemically functionalized to enable the immobilization of the bioreceptor. The chemical functionalization of graphene can be also be used to tailor the electronic properties of graphene via doping and band-gap engineering effects, produced by chemical modification or adsorption of molecules on to the graphene [18].

The functionalization of graphene with a linker molecule can be performed through covalent binding to the carbon atoms of the hexagonal matrix or by non-covalent binding to the graphene by electrostatic and/or weak Van der Waals forces [18]. A wide range of potential functionalization chemistries, such as halogenation, hydroxylation, epoxidation, carboxylation, amination, alkylation, and azidation, have been developed for graphene [27]. The presence of *sp^2^* carbon atoms makes the graphene surface a potential candidate for covalent bonding [28]. Covalent chemistries used to make graphene functional include fluorination [29] and hydrogenation [30] by plasma treatments. Also utilized is free-radical addition to the carbon atoms of the hexagonal matrix [31], such as diazotization [32]. Other covalent methods include the covalent attachment of polymers such as PEG [31] and silanization by 3-aminopropyltriethoxysilane (APTES) [33]. Tehrani et al. demonstrated the development of a G-FET for cancer risk biomarker (8-OHdG) with a limit of detection of 0.1 ng·mL^−1^ using the diazonium functionalization chemistry [34]. Teixeira and co-workers reported the detection of human chorionic gonadotropin (hCG) at 0.62 ng·mL^−1^ using an epitaxial G-FET functionalized using the APTES method [33]. Although covalent chemistry has proven to be successful, it also creates undesirable disruption to the *sp^2^* nature of the carbon atoms. As a result, the *sp^2^* hybridization will be converted to *sp^3^* hybridization [28], which disrupts the electron structure of graphene, and therefore diminishes the excellent and desirable electronic properties of graphene. Therefore, other avenues of graphene functionalization have been explored [18].

Non-covalent functionalization is dominated by the physisorption of molecules to the graphene through weak Van de Waals forces [18]. More specifically, this non-covalent functionalization often occurs through an interaction between the π-electron cloud of the graphene and the functional molecule, otherwise known as π–π stacking. Graphite (bulk graphene) is an example of π–π interaction. Graphite is multiple layers of graphene sheets stacked upon one another through an interaction between their respective π-electron clouds [31]. Since this non-covalent functionalization of graphene occurs in this way, the *sp^2^* nature of the carbon atoms is not affected. Therefore, the electronic and structural properties are not severely disrupted [18], making this a desirable method of functionalization for G-FET biosensor development. Often, the molecule used for functionalization has a polyaromatic hydrocarbon base, such as benzene, naphthalene, or pyrene, with pyrene exhibiting a strong affinity towards graphene through π-stacking [35]. Chen et al. demonstrated the effect of some of these electron withdrawing and electron donating molecules on the graphene’s electronic properties. It was reported that functionalization with tetrafulvalene (TTF), an electron donor, acts to p-dope the graphene, whilst an electron acceptor, hexaazatriphenylene-hexacarbonitrile (HATCN), acts to n-dope the graphene. However, both remained non-destructive to the graphene’s electronic and structural properties [18]. Furthermore, functionalizing the graphene surface using a pyrenebutanoic acid succinimidyl ester (PBASE) through π-stacking is attractive, as the pyrene base of this molecule exhibits a strong affinity to the graphene sheet, whilst the succinimidyl ester provides a binding site for amines of various biomolecules, including antibodies, enzymes, bacteria, and nucleic acid probes [25,36,37,38,39,40]. Moreover, several non-covalent functionalization techniques have been developed to decorate the graphene surface using metal nanoparticles, such as gold [41], platinum [42], palladium [43], and zinc oxide [44]. Metal nanoparticles can be deposited onto the graphene channels by immersing the channel into the metal salt solution, electrochemical deposition, or by a chemical reduction process. Gutes et al. reported that the nature of the metal dictates the size and densities of the as-prepared metal nanoparticles, despite the same experimental conditions. For example, platinum metal appeared to form smaller particles with lower density when compared to gold and palladium [43]. Cai et al. utilized gold nanoparticles on a G-FET to create a binding site for a sulphur-terminated biorecognition molecule. Moreover, Cai et al. reported the presence of nanoparticles to increase the active surface area of the G-FET, which in turn improved the sensitivity by providing more binding sites for biomolecule immobilization [41]. 

## 3. G-FET-Based Nucleic Acid Sensors

**Figure 2:**
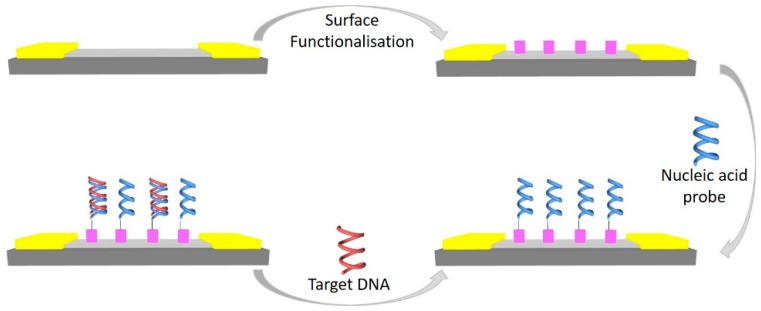
A schematic representation of the process flow for developing a G-FET for nucleic acid detection. Gold—Contact pads, dark grey—SiO_2_, light grey—graphene, purple—surface functionalisation.

Nucleic acids such as deoxyribonucleic acid (DNA), ribonucleic acid (RNA), and microRNA (miRNA) play a major role in human physiology, and therefore they also play a major role in many diseases. As a result, rapid and highly sensitive detection methods of nucleic acid abnormalities or expression are considered extremely important for disease diagnosis [39]. G-FETs for DNA tend to be more sensitive and therefore more responsive to target analytes than the widely researched and developed ion-sensitive FET, which is attributed to the difference in the sensing mechanisms. Nucleic acids in close proximity with the graphene surface, whether physisorbed or through hybridization events, considerably change the graphene’s electronic properties by doping the graphene. This causes a direct change to the graphene’s properties. The standard bulk ion-sensitive FET, however, responds to changes in external charges, which cause a change in the channels’ capacitive properties [45]. In the case of nucleic acid-based biosensors, the biorecognition molecule is often a nucleic acid probe, as depicted in Figure 2.

### 3.1. DNA Sensor

DNA, a double stranded polynucleotide, contains the entire genetic code of an individual, therefore assessing an individual’s genetic makeup can not only aid in the diagnosis of many diseases, but also contains information regarding an individual’s predisposition to genetic diseases and cancers. The DNA nucleotide is made up of a phosphate group, which makes the backbone of the DNA polynucleotide, a sugar (2-deoxyribose), and a nucleobase (adenine = A, guanine = G, thymine = T, and cytosine = C). These nucleotides arrange in specific sequences through phosphodiester bonding between nucleotides to make up the genome, which stores and transmits genetic information. A complementary strand of DNA then binds via the hydrogen bonding of the nucleobases (A with T and G with C) to make it a double-stranded helix [49]. Since DNA contains important genetic information, it is highly important to develop rapid, specific, and sensitive methods of detection for DNA. Developing such tests will aid considerably in disease diagnosis, genetic screening [41], pharmacogenomics, molecular diagnostics, drug discovery, and potentially prevention by enabling early treatment [25].

**Figure 3:**
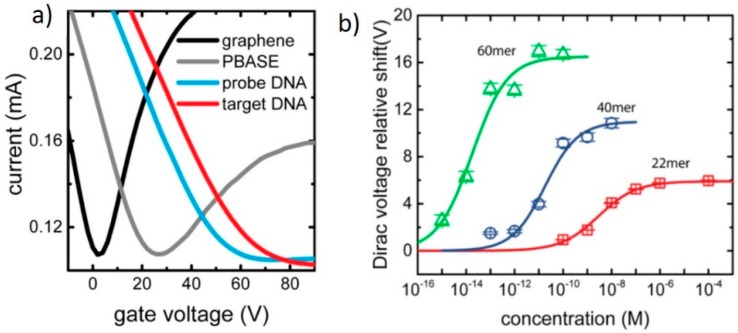
(**a**) I-V_g_ characteristics of a G-FET proposed by Ping et al., highlighting the change in electronic characteristics at each stage of the functionalization and detection process. (**b**) Dirac voltage shift of increasing concentrations of DNA oligomers of different lengths fitted using the Sips model. Adapted from [25]. Copyright 2016 by the American Chemical Society.

Over the past decade, several biosensor techniques have been developed for the high sensitivity detection of DNA. Several G-FET-based DNA biosensors have been developed using various sensing methods, including electrochemical [50], back-gated G-FETs [25], and liquid-gated G-FETs [45]. DNA-based G-FETs follow conventional DNA detection mechanisms. Short DNA oligomers (DNA probes) are used for biorecognition. DNA probes are short nucleotides which are complementary to the target DNA. These DNA probes are either immobilized to the sensor surface and act as a capture probe for the target DNA [38,51], or they are tagged and bound secondarily to target DNA captured on the sensor surface [50]. Alternatively, DNA can also be detected by physisorption, as the nucleobases which make up DNA are aromatic carbons, and thus are able to bind to the graphene via π-stacking [52]. Ping et al. demonstrated a scalable (>90% yield) back-gated G-FET DNA biosensor with 1 fM sensitivity for a 60-mer DNA. The G-FET was fabricated by transferring CVD-grown graphene onto a pre-fabricated SiO_2_ substrate with 45 nm thick Cr/Au contacts by an electrolysis bubbling method. Using a PBASE linker, the 22-mer DNA probe was attached to the graphene surface. It was reported that the Dirac peak of the graphene shifted increasingly at each stage of functionalization (highlighted in Figure 3) and furthermore with increasing DNA concentration. Ping et al. also confirmed the high selectivity of the probe by applying a single-nucleotide mismatched DNA strand and a non-complementary DNA strand. The application of the one-base mismatched DNA to the sensor resulted in a signal change only 12% of that of the complementary DNA [25].

**Figure 4:**
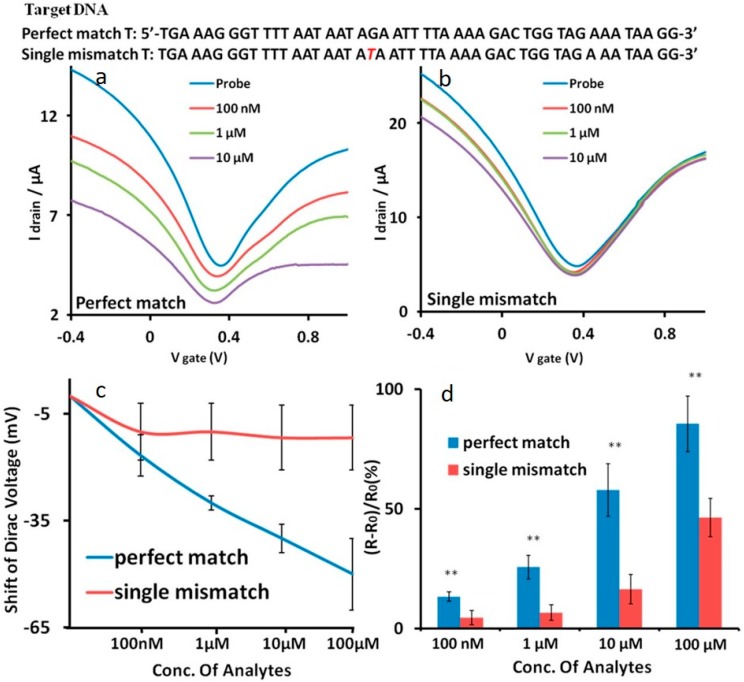
Transfer curves for a G-FET produced by Hwang et al. for each stage of the strand displacement sensing process for (**a**) perfect match DNA and (**b**) single nucleotide polymorphism (SNP) DNA. (**c**) V_D_ shift for perfect match and SNP DNA at various concentrations showing the clear discrimination between perfectly matched DNA and DNA containing an SNP. (**d**) Quantitative measurement of both the perfectly matched DNA and DNA containing an SNP using resistance changes across the graphene channel. For the data highlighted here ** *p* < 0.01 based on three sets of independent data points. Reprinted from [38].

Many genetic-related diseases are caused by an abnormality in DNA expression or genetic information. Therefore, it is not only important to develop sensors to detect aberrant expression but also abnormalities in the genetic code. Abnormalities exist as mutations in the genetic sequence. The most common of these mutations is known as a single nucleotide polymorphism (SNP), otherwise known as a single nucleotide mutation in the DNA sequence [53]. These mutations can have a dramatic effect on an individual’s health. SNPs have previously been reported to be involved in the development of cancers and genetic disorders. Hwang and co-workers have reported the development of a highly specific and sensitive SNP detection using a G-FET. The G-FET reported in this work acted on a strand displacement principal, which is a method employed widely across the medical profession. A double-stranded DNA (dsDNA) probe was immobilized upon the CVD-based G-FET surface via PBASE. One strand of this dsDNA is the complementary sequence to the target DNA. The second strand was essentially the same sequence as the target DNA; however, four guanine bases were substituted with inosine bases to weaken the binding affinity between the two strands. On exposure of the G-FET to the target DNA containing an SNP and the perfect match DNA, the inosine modified strand was displaced. The perfectly complementary target DNA exhibited a V_D_ shift of −50 mV by n-doping for 100 µM DNA and −11.6 mV for 100 µM target DNA containing an SNP. Hwang et al. demonstrated a G-FET which can discriminate the target DNA and DNA containing an SNP. This discrimination was reported to be possible over a range of concentrations, from 100 nM to 100 µM, as highlighted in Figure 4a–c. In addition, a direct quantification of each target DNA type was illustrated by a change in the resistance of the graphene channel, as depicted in Figure 4d [38].

**Figure 5:**
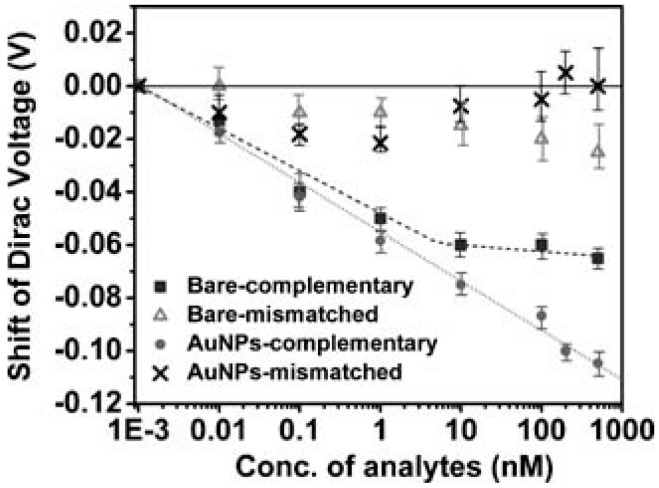
Shift in V_D_ for the bare electrode and AuNP decorated G-FET when adding increasing concentrations of complementary DNA and one-base mismatched DNA. Reprinted from [54]. Copyright 2010 by John Wiley and Sons.

An important factor in designing a molecular biology test is sensitivity and the linear dynamic range. Both can be influenced by many factors, including but not limited to: graphene quality, a probe’s affinity for the target, the efficiency of hybridization and the surface coverage of the capture probe, and the surface-to-volume ratio. Although graphene inherently has an extremely high surface-to-volume ratio, it can still be a limiting factor when improving the sensitivity of the G-FET. However, it is not impossible to enhance the surface-to-volume ratio further, and, as a result, the sensitivity [41]. Dong et al. demonstrated this enhancement in surface-to-volume ratio, and, as a result, the linear dynamic range. Two CVD on Si substrate G-FETs were developed. The DNA probe was immobilized to the graphene surface through a π-stacking interaction on one G-FET, which was named the bare electrode. The other was decorated with gold nanoparticles, and a thiolated DNA probe was immobilized. Both G-FET devices were then exposed to varying concentrations of target DNA: it can be seen in Figure 5 that the bare electrode showed a dynamic range of 10 pM to 10 nM before saturation. The Au nanoparticle (AuNP)-decorated G-FET, however, extended the upper limit of detection to 500 nM, 50-fold of the upper limit of the bare electrode, suggesting an enhanced sensitivity and detection by decorating the graphene with AuNPs [54].

### 3.2. miRNA Sensor

**Figure 6:**
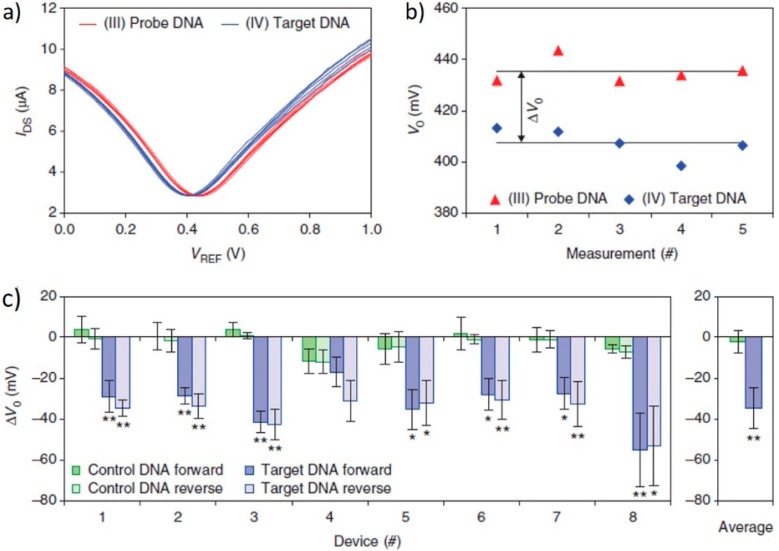
(**a**) Dirac (V_g_–I_DS_) curves for a single G-FET device before and after 100 fM miRNA target exposure. A total of five forward and reverse sweeps were performed on a single device. (**b**) V_D_ values for each sweep calculated from the graphs depicted in (**a**). (**c**) ∆V_D_ values noted for all eight G-FETs on exposure to the target DNA sequence and to the control DNA sequence. It can be noted that the response seen for the control DNA is considerably less than that caused by the target DNA. For the data highlighted here: * *p* < 0.05; ** *p* < 0.01, not significant. Adapted by permission from Macmillan Publishers Ltd.: [45], copyright 2014.

MicroRNAs are short chain RNAs consisting of approximately 22 nucleotides. miRNAs have previously been reported to be closely related to many diseases, including cancer. The link between the development and pathogenesis of these diseases and miRNAs has been said to occur when the miRNA expression deviates away from the normal standard [55]. miRNAs are encoded within the genome and act to downregulate gene expression, a role which is vital for the homeostasis of the human body. miRNAs downregulate gene expression by either one of two methods: mRNA cleavage or translational repression. Since the role of miRNA in maintaining normal levels of gene expression is vital to human physiology and function, a deviation away from this leads to disease development, including human cancers [56]. In 2014, Xu et al. [45] demonstrated the successful development of a G-FET specific for let7g, a miRNA widely believed to play a role in tumour suppression. Xu et al. produced the G-FET using CVD graphene transferred onto a SiO_2_/Si substrate with Cr/Au/Cr contacts, and applied a polydimethylsiloxane (PDMS) microfluidic to avoid contact interference in the signal. Using the well documented streptavidin-biotin binding mechanism, the 41-mer DNA probe was immobilized upon the graphene surface. A biotinylated bovine serum albumin (BSA) was absorbed onto the channel, and streptavidin was then bound to that biotinylated BSA. The DNA probe (also biotinylated) was then introduced, and was able to immobilize upon the channel via binding to one of the three remaining binding sites on the streptavidin. On exposing the eight G-FETs to 100 fM of the target miRNA, a noticeable negative shift was observed due to the electron doping effect of DNA hybridization on the graphene channel. Xu et al. also confirmed the selectivity of their devices by applying a control nucleotide to the sensor. The response seen for the control nucleotide was negligible when compared to the response exhibited by the hybridization event, which occurred between the probe and the target miRNA (highlighted in Figure 6) [45].

**Figure 7:**
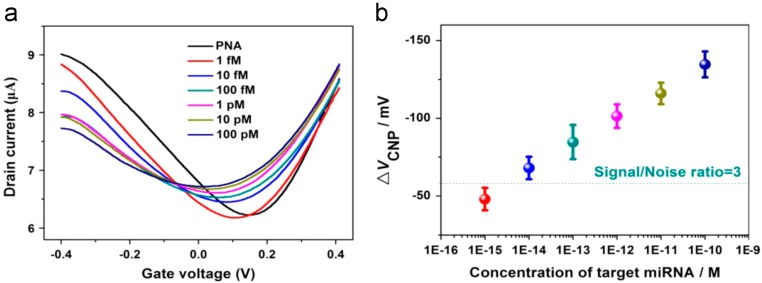
(**a**) The transfer curve of a G-FET decorated with AuNPs with immobilized PNA probe when exposed to increasing concentrations of Let7b miRNA. (**b**) It can be noted that V_D_ progressively decreases in V_g_ due to an n-doping effect of the graphene by miRNA hybridization. Reprinted from [41], Copyright 2015, with permission from Elsevier

Cai et al. demonstrated enhanced sensitivity by addressing two of the previously mentioned influential factors on G-FET sensitivity. Firstly, Cai et al. exchanged the DNA probe for a peptide nucleic acid (PNA) probe. PNA is essentially the same as DNA, however due to the exchange of the deoxy-ribose and phosphate backbone for a peptide backbone it is essentially a neutral form of DNA. However, PNA is still able to exhibit an effect on the graphene’s doping due to the electron-rich nucleobase. Therefore, it is still possible to note a shift in the V_D_ of the graphene. The advantage of using a PNA probe, as reported by Cai and co-workers, was the diminished repulsion between DNA molecules caused by the negative backbones of the DNA, therefore enhancing the hybridization’s efficiency. Secondly, Cai et al. reported a G-FET decorated with AuNPs with a lower limit of detection of 1 fM (highlighted in Figure 7). The PNA probe was immobilized onto the AuNPs by a cysteamine and glutaraldehyde binding step. Decorating the graphene surface with AuNPs reportedly improved the sensitivity by 1 order of magnitude when compared to a G-FET which was not decorated with AuNPs. The improvement noted in sensitivity was attributed to the significant increase in surface area from the addition of the AuNPs [41].

## 4. Immunosensors

**Figure 8:**
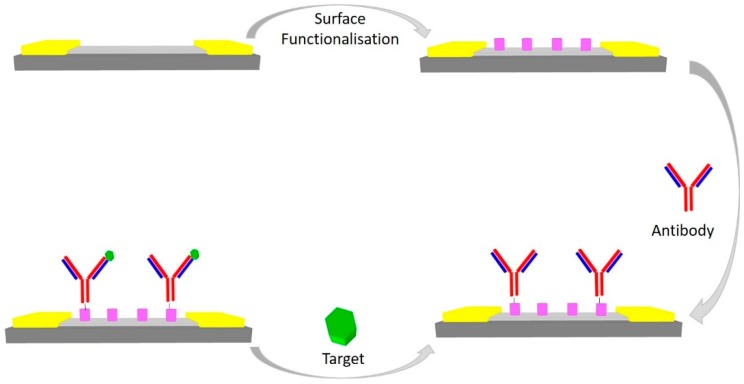
A schematic representation of the process flow for developing an immuno-based G-FET. Gold—Contact pads, dark grey—SiO_2_, light grey—graphene, purple—surface functionalisation.

Immunoassays are biomolecular recognition tests commonly used to determine the presence of biomarkers in a solution and potentially quantify them. More specifically, immunoassays are analytical techniques which rely on biorecognition by antibody-antigen interactions. Therefore, the techniques are based on the specificity and affinity of the antibody for the respective antigen [57]. Immunosensors are developed by the immobilization of an antibody onto the G-FET’s surface. Detection then occurs when the target analyte binds to the antigen binding fragment of the antibody, as depicted in Figure 8. Many G-FETs for immunoassays have been reported in the literature [33,34,46,47].

**Figure 9:**
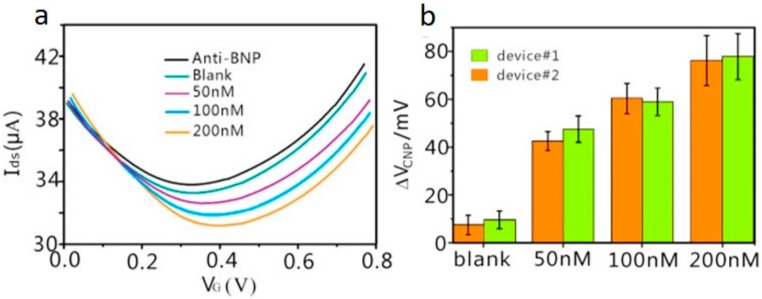
(**a**) Transfer curves of a PtNPs-decorated rGO-FET in response to brain natriuretic peptide (BNP) in whole blood samples which have been treated with a microfilter; (**b**) Dirac voltage shift in response to the differing concentrations of BNP. Adapted from [46], Copyright 2017, with permission from Elsevier.

Lei and co-workers reported the successful detection of a protein biomarker in whole blood, which is specific to heart failure, using a platinum nanoparticle (PtNP) decorated rGO-FET immunosensor technology. The binding of brain natriuretic peptide (BNP) to anti-BNP was able to be detected through liquid gated measurements at 100 fM. Adding to this, the BNP was able to be distinguished from whole blood proteins, namely, human serum albumin and D-Dimer. Furthermore, BNP was successfully detected in a whole blood sample treated with a microfilter, reported in Figure 9. This indicated that the immunosensor was capable of distinguishing BNP from other proteins within the complicated sample matrix of whole blood [46].

**Figure 10:**
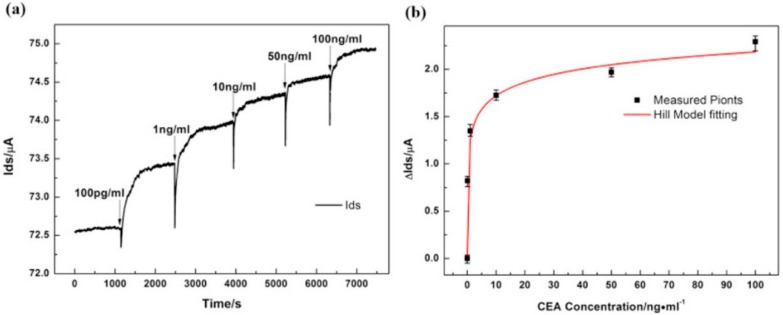
(**a**) Drain-source current response at the time-dependent introduction of various carcinoembryonic antigen (CEA) concentrations; (**b**) Drain-source current against CEA concentration fitted based on Hill adsorption model. Adapted from [47], Copyright 2017, with permission from Elsevier.

In 2017, Zhou et al. demonstrated the development of a G-FET for the real-time monitoring of carcinoembryonic antigen (CEA) detection, a biomarker for cancer. Zhou and co-workers reported a detection limit of 100 pg/mL (0.5 pM), far exceeding that of the clinical diagnostics cut-off value. Anti-CEA was immobilized to the G-FET through PBASE, and subsequently the binding of CEA was detected by chronoamperometry. An increase in the drain current was observed, correlating with increasing CEA concentration, as depicted in Figure 10 [47].

### Debye–Hükel Screening

**Figure 11:**
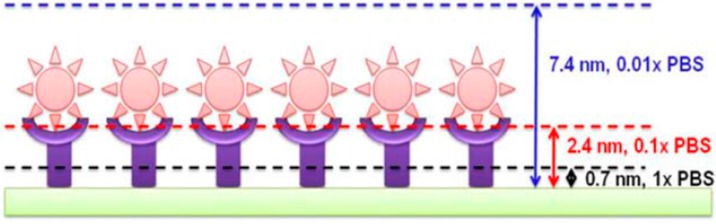
An illustration highlighting how different ionic buffer solution concentrations affect the screening length (λ_D_). Green—sensor platform, purple—bioreceptor, pink—antigen. Reprinted from [17], Copyright 2011, with permission from Elsevier.

Even though many immuno-based sensors have been reported in the literature, it is still challenging to reach ultra-high sensitivities because of Debye–Hükel screening [52]. Debye–Hükel screening is a phenomenon caused by the solution’s interaction with the sensor [17]. Ionic solutions effectively screen the charge of analytes in proximity with the sensor surface by forming an electron double-layer. The length at which the analyte is able to be screened, otherwise known as the Debye screening length (λ_D_), is highly dependent on buffer concentration [52]. Therefore, immunoFET detection is essentially limited to interactions which occur within a small distance of the electrode surface. Molecules outside of the λ_D_ are generally unable to be detected, as the charges within the graphene channel are unaffected [58]. As depicted in Figure 11, the λ_D_ decreases with increasing buffer concentration [17]. The Debye screening phenomenon makes it difficult to develop a highly sensitive immunosensor, as high ionic strength buffer solutions are required for biological species, therefore decreasing the λ_D_, and making it difficult to use antibodies as the capture molecule [52].

This issue has been addressed by many by only utilizing the antigen binding fragment (Fab) of the antibody. This decreases the distance of the antigen antibody interaction from the surface from approximately 10–15 nm for the whole antibody to approximately 3–5 nm for the Fab, allowing for the use of higher ionic strength buffers [16,59]. Many have also addressed the Debye screening phenomenon through the development of aptasensors [36,48,58,60,61]. Aptamers are short chain peptides or single-stranded nucleic acids designed to fold into a three-dimensional (3D) structure specifically for binding target analytes. Aptamers have attracted considerable attention due to their ease of synthesis, high binding efficiency and affinity, specificity, and high stability. Most of all, aptamers have been extensively researched due to their small size (less than 5 nm), which is a desirable trait to combat the issues faced with Debye screening [14]. Both Saltzgaber and co-workers and Wang et al. reported the successful detection of thrombin, a cardiovascular biomarker, using the aptamer based G-FET approach [36,61]. Others have reported the detection of vascular endothelial growth factor (VEGF), a tumour growth and metastasis biomarker [60], and bisphenol A (BPA) (a chemical found in packaging which is known to be hazardous to human health) [62].

**Figure 12:**
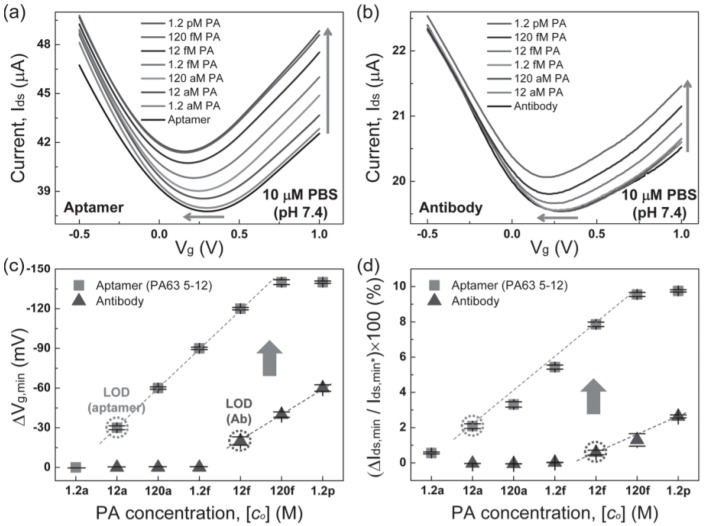
Transfer curves of a G-FET with increasing concentrations (depicted by the arrows) of protective antigen (PA) using an (**a**) aptamer and (**b**) antibody. These were then depicted as (**c**) Dirac voltage shift and (**d**) change in drain-source current. Reprinted from [48], Copyright 2013, with permission from John Wiley and Sons.

Kim et al. reported research addressing this issue. The research directly compared the performance of an aptamer-based G-FET and an antibody-based G-FET for protective antigen (PA), a target analyte for detecting anthrax. A single-stranded DNA aptamer (PA65 5–12) and anti-PA were used. A comparison of the range of detection, sensitivity, and limit of detection proved the aptamer-based sensor to have an overall better performance to the antibody based sensor, as depicted in Figure 12. The aptasensor had a detection range of 12 aM to 120 fM, with a sensitivity of 30 mV/decade, whilst the antibody-based sensor exhibited a detection range of 12 fM to 1.2 pM, with a sensitivity of 20 mV/decade. This indicated that the limit of detection had dropped 3 orders of magnitude when using the aptamer sensor as well as improving the detection range by 2 orders of magnitude [48]. These results were supported by the less sensitive detection of PA previously reported, which showed an antibody-based G-FET with a limit of detection of 1 fM [63].

## 5. Current Challenges and Future Prospects

For over a decade, considerable scientific effort has been directed towards the development of G-FETs for biosensing applications. This review highlights the recent developments in G-FET biosensors, with an emphasis on nucleic acid-based sensors. Label-free G-FETs have shown sensitivities as low as attomolar, far lower than those usually exhibited by other semiconductor technologies or current bioanalytical methods, attesting to G-FET biosensors as a potential platform towards clinical applications. There are, however, challenges faced in the development of G-FET biosensors. One of these limitations is device sensitivity due to the Debye–Hükel phenomenon and limited surface area. These issues were highlighted in Section 4 of the review with examples.

The Debye–Hükel phenomenon becomes a hindrance in developing highly sensitive G-FET biosensors, as high ionic strength buffers are needed for the analyte solutions. This decreases the Debye screening length, and as a result decreases the sensitivity of the G-FET to target analytes outside of this length. Therefore, although the field of G-FET technologies is rapidly improving, the development of immunoFETs is hindered by Debye-screening. However, significant R&D efforts have focused on bypassing this issue through the development of nucleic acid-based sensors, aptasensors, and antigen binding fragment (Fab) modified G-FETs. The use of aptamers and Fabs as biorecognition molecules decreases the distance of the interaction from 10–15 nm to 3–5 nm, well within the debye-screening length of 7.4 nm that is seen for 0.01× PBS solution. The development of aptamers and Fabs have led to a biorecognition technology which can replace antibodies and will possibly drive forward the development of immunoFET technologies.

The second issue is the surface area of the G-FET sensor. Although graphene has an inherently high surface area, it was reported that this feature could be further improved, and as a result the sensitivity could be increased. This was possible through decorating the G-FET surface with metal nanoparticles, increasing the binding sites for the biorecognition element, and therefore the target analyte.

It is clear that graphene has many superior qualities when compared to other semi-conductor technologies. However, the majority of these measured characteristics and aforementioned G-FET sensors have only been achieved using the highest of quality samples within a laboratory setting. To date, most of the work has focused on R&D efforts, as although rapidly improving, these exceptional properties still remain difficult to obtain in a mass-scale manufacturing process [64]. Deokar et al. demonstrated the high quality growth of CVD graphene that was free of residue and contamination, which is a vital aspect needed for moving graphene-based biosensors from the lab to industry. It is the status of these large-scale production processes which are the driving force behind the development of graphene for commercialization [22]. Furthermore, the scalability of these processes also remains a bottle-neck in production. However, once a “gold-standard” is reached, a growing interest in graphene for commercialization will most likely be observed. Many challenges will need to be faced in the commercialization of G-FETs, for example identifying routes to incorporate G-FETs into existing technologies or commercial systems, and eventually the replacing the existing technologies with these new concepts [64]. The Graphene Flagship initiative aims to develop consumer products from graphene by 2025–2030. The initiative describes the process of graphene commercialization as a hierarchy of many stages. These are understanding its properties and processes, device concepts and proof of principle, technologies for quality wafer-scale manufacturing, prototypes, viable technologies, and finally products. At present, graphene commercialization is in the device concept and proof of principle stage, with few prototypes having been developed [65]. To move the development of G-FETs forward, the proof-of-concept devices must be developed further into the prototype stage. This has to be done by moving from testing using buffered solutions to testing the analytes in situ. Many of the nucleic acid biosensors have been developed using synthesized short chain nucleotides. Moving forward, longer chain nucleotides or whole genes must be considered to enable the G-FETs developed to be applicable in clinical settings. Nonetheless, G-FETs promise to bring new and exciting alternatives to current healthcare diagnostics.

Furthermore, to develop efficient G-FET biosensors with high accuracy, precision, reproducibility, and lower detection limits, it is vital to improve the biomolecular immobilization strategies. Therefore, more functionalization chemistries need to be identified. The exploration of various bioreceptors, such as aptamers and antibody fragments, would certainly increase their sensitivity. Moreover, the nano-bio interfaces in G-FET sensors should be investigated in more detail. The real-time detection and stability of such sensors also needs to be analyzed in detail to enable the commercialization of G-FET biosensors that exhibit long-term stability and superior performance for clinical practice.

## Figures and Tables

**Table 1:** A list of graphene-based field effect transistor (G-FET) biosensors currently reported in the literature.

Type of Sensor	Target	Application	Substrate	Detection Method	Detection Limit	Control	Signal-to-Noise	Ref.
Nucleic acid	22-mer DNA	Proof-of-concept	2 × 2.5 cm CVD graphene on SiO_2_, Cr/Au contacts	Back gated, DNA probe	100 pM	One-base mismatched	-	[25]
	20-mer DNA	Proof-of-concept	45 × 90 µm CVD on SiO_2_/Si, Cr/Au contacts	Liquid gated, DNA probe	10 pM	One-base mismatched	-	[37]
	22-mer DNA	Proof-of-concept	4 µm CVD graphene channel on SiO_2_/Si, Ti/Au contacts	Liquid gated, PNA probe	10 fM	One-base mismatched, non-complementary	3	[39]
	22-mer miRNA (Let7g)	Cancer	45 × 90 µm CVD on SiO_2_/Si, Cr/Au/Cr contacts	Liquid Gated. RNA probe	100 fM	Non-complementary miRNA	-	[45]
	22-mer miRNA (Let7b)	Cancer	rGO on SiO_2_/Si, Decorated with Au nanoparticles (AuNPs)	Liquid gated, PNA probe	1 fM	One-base mismatched and non-complementary	3	[41]
Immunosensor	Brain natriuretic peptide (BNP)	Heart failure	rGO on SiO_2_/Si, Decorated with PtNPs	Liquid gated, Anti-BNP	100 fM	BSA, D-Dimer, and HSA	3	[46]
	Carcinoembryonic antigen (CEA)	Cancer	25 × 50 µm CVD on SiO_2_/Si, Ti/Au contacts	Liquid-gated, Anti-CEA	0.5 pM	-	-	[47]
	Human Chorionic Gonadotropin (hCG)	Pregnancy	Epitaxial on SiC, Ti/Au contacts	I-V, Anti-hCG	16.7 pM	Urea and Cortisol	-	[33]
	8-hydroxydeoxyguanosine (8-OHdG)	Cancer	250 µm × 3 mm Epitaxial on SiC, Ti/Au contacts	I-V, Anti-8-OHdG	0.35 nM	PBS no 8-OHdG	-	[34]
	Protective antigen (PA)	Anthrax	GO nanosheets on glass, Ti/Au contacts	Liquid gated, PA65 5–12 aptamer	12 aM	-	-	[48]

rGO—reduced graphene oxide, PNA—peptide nucleic acid, BSA—bovine serum albumin, HSA—human serum albumin.
